# Key Drivers and Facilitators of the Choice to Use mHealth Technology in People With Neurological Conditions: Observational Study

**DOI:** 10.2196/29509

**Published:** 2022-05-23

**Authors:** Sara Simblett, Mark Pennington, Matthew Quaife, Evangelia Theochari, Patrick Burke, Giampaolo Brichetto, Julie Devonshire, Simon Lees, Ann Little, Angie Pullen, Amanda Stoneman, Sarah Thorpe, Janice Weyer, Ashley Polhemus, Jan Novak, Erin Dawe-Lane, Daniel Morris, Magano Mutepua, Clarissa Odoi, Emma Wilson, Til Wykes

**Affiliations:** 1 Psychology Department King's College London London United Kingdom; 2 Health Economics Department London School of Hygiene & Tropical Medicine London United Kingdom; 3 Neurosciences Department King's College Hospital London United Kingdom; 4 Remote Assessment of Disease and Relapse in Central Nervous System Disorders Patient Advisory Board King's College London London United Kingdom; 5 Italian Multiple Sclerosis Society and Foundation Rome Italy; 6 International Bureau for Epilepsy Dublin Ireland; 7 Epilepsy Action Leeds United Kingdom; 8 Merck Sharp & Dohme Information Technology Prague Czech Republic; 9 Faculty of Science Charles University Prague Czech Republic; 10 South London and Maudsley Biomedical Research Centre London United Kingdom

**Keywords:** mobile technology, neurological conditions, multiple sclerosis, epilepsy, discrete choice experiment, digital health, mHealth, wearable technology, wearable biosensors, health economics, health data

## Abstract

**Background:**

There is increasing interest in the potential uses of mobile health (mHealth) technologies, such as wearable biosensors, as supplements for the care of people with neurological conditions. However, adherence is low, especially over long periods. If people are to benefit from these resources, we need a better long-term understanding of what influences patient engagement. Previous research suggests that engagement is moderated by several barriers and facilitators, but their relative importance is unknown.

**Objective:**

To determine preferences and the relative importance of user-generated factors influencing engagement with mHealth technologies for 2 common neurological conditions with a relapsing-remitting course: multiple sclerosis (MS) and epilepsy.

**Methods:**

In a discrete choice experiment, people with a diagnosis of MS (n=141) or epilepsy (n=175) were asked to select their preferred technology from a series of 8 vignettes with 4 characteristics: privacy, clinical support, established benefit, and device accuracy; each of these characteristics was greater or lower in each vignette. These characteristics had previously been emphasized by people with MS and or epilepsy as influencing engagement with technology. Mixed multinomial logistic regression models were used to establish which characteristics were most likely to affect engagement. Subgroup analyses explored the effects of demographic factors (such as age, gender, and education), acceptance of and familiarity with mobile technology, neurological diagnosis (MS or epilepsy), and symptoms that could influence motivation (such as depression).

**Results:**

Analysis of the responses to the discrete choice experiment validated previous qualitative findings that a higher level of privacy, greater clinical support, increased perceived benefit, and better device accuracy are important to people with a neurological condition. Accuracy was perceived as the most important factor, followed by privacy. Clinical support was the least valued of the attributes. People were prepared to trade a modest amount of accuracy to achieve an improvement in privacy, but less likely to make this compromise for other factors. The type of neurological condition (epilepsy or MS) did not influence these preferences, nor did the age, gender, or mental health status of the participants. Those who were less accepting of technology were the most concerned about privacy and those with a lower level of education were prepared to trade accuracy for more clinical support.

**Conclusions:**

For people with neurological conditions such as epilepsy and MS, accuracy (ie, the ability to detect symptoms) is of the greatest interest. However, there are individual differences, and people who are less accepting of technology may need far greater reassurance about data privacy. People with lower levels of education value greater clinician involvement. These patient preferences should be considered when designing mHealth technologies.

## Introduction

Mobile health (mHealth) technologies such as wearable biosensors could supplement the care of people with neurological conditions, as symptoms of disability can evolve over time and are generally hard to capture through single measurements. mHealth technologies can help to detect variations in movement and physiological signals that indicate changes in the underlying disease state, thereby allowing earlier intervention or tailored therapy. While there is emerging evidence to suggest that mHealth technologies are acceptable to people with neurological conditions, such as epilepsy, stroke, multiple sclerosis, dementia, and Parkinson’s disease [1-6], adherence, especially over long periods, can be low. In a recent systematic review of engagement with remote measurement technology (RMT) for health care support, we found that despite studies being short (the longest study was 13 months) they had variable, and in some cases relatively high, dropout rates (0%-44%) [7]. If people with neurological conditions are to benefit from these resources over the long term, we need a better understanding of what influences their engagement.

Theories of technology engagement emphasize the role of beliefs and perceptions [8]. In general, when there is motivation to use mHealth technologies, and these tools are perceived to be useful, accessible, and convenient, user engagement may be high [7,9]. Specifically, factors such as performance expectancy (how much technology will help to achieve something); effort expectancy (how easy technology is to use); and how well resourced and supported the technology is, account for differences in behavioral intentions and affect mHealth technology use (with a medium effect size) [10]. However, there are additional factors that are correlated with variability in mHealth technology use, including demographic variables, such as gender and age [11,12], prior experience with technology [13], and social influence (how important the technology is to others) [8]. People with different diagnoses also have different views of what might attract or deter them from RMT use. In this study, we have chosen to focus on 2 specific neurological conditions in which people experience a relapsing-remitting course and for which there is already some evidence that technology may be an acceptable method for long-term symptom management: multiple sclerosis (MS) and epilepsy. In our earlier work, we identified barriers, facilitators, and moderators specific to people with MS or epilepsy [4-6]. For both conditions, we highlighted the need to balance costs against rewards when deciding on the use of mHealth technologies. Better understanding these trade-offs would provide a more sophisticated understanding of which factors most influence engagement in these two populations. This would enable us to develop technology that is more acceptable to people with neurological conditions such as MS and epilepsy, encouraging long-term adherence.

One approach emerging from health care economics is the use of discrete choice experiment (DCE) surveys [14,15]. DCEs have been used to break down health care interventions or services into characteristics, known as attributes, and then to quantify their relative value by asking individuals to choose between services described according to varying attribute levels. Analysis of these choices allows the identification of the most important and preferred attributes [14,15]. Previous DCEs have explored preferences for health care choices among people with epilepsy or MS, and have investigated factors such as the design of interventions, (eg, whether people with MS prefer oral treatment versus intravenous infusions) [16-19], methods of diagnosis (eg, if people with epilepsy prefer long-term 24-hour electroencephalography or sleep-deprived electroencephalography) [20], the best targets for treatment (eg, whether patients prefer to delay the progression of their disability or improve their quality of life) [21,22], and the acceptability of different medications (such as by comparing different side effects) [23-27]. Meta-analyses have shown that DCEs can produce reasonable predictions of real-world health-related behaviors [28]. Ryan et al [29] argue that the strength of this approach lies in the integration of patients’ values concerning all aspects of care into a single measure; this could inform the efficient allocation of resources in a health care system, particularly in relation to the introduction of new technologies.

Research on relative preferences for sharing health data, which is integral to digital technology use, has discovered that information on mental health is more sensitive than information on physical health, and that privacy-utility trade-offs are important [30]. The present study extends these findings, to explore the relative importance of several other dimensions that have been shown to affect mHealth technology engagement in people with MS or epilepsy, and determined whether any of these dimensions varied by subgroup. We chose to investigate potential moderators identified in previous research on the general public and specific patient populations: demographic factors (such as age, gender, and education), acceptance of and familiarity with mobile technologies, and symptoms that could affect motivation (such as depression).

## Methods

### Study Design

This was an observational study of participants with MS or epilepsy. The participants were asked to choose between alternative mobile technologies that were described according to a set of characteristics in a DCE survey. The survey was administered online and was given ethical approval by the National Research Ethics Service Committee London—Social Care (19/IEC08/0013).

### Development and Implementation of the DCE

#### Service Users’ Identification of Key Barriers and Facilitators for mHealth Technology Engagement and Assignment of Levels

A systematic review was used to generate 8 potential attributes that could vary continuously [7]. This list of attributes was checked against data from a qualitative analysis of 9 focus groups, including 44 people who had received a diagnosis of either MS [6] or epilepsy [5], and an analysis of a further 5 focus groups, including 25 people who reported symptoms of depression (which are commonly comorbid with neurological conditions) [31]. After this review, 7 more attributes were added, for a total of 15. The final list of 15 attributes and levels was sent to a patient advisory group, which included 5 people with epilepsy, 3 people with MS, and 6 people with depression. This group independently ranked the list in order of importance. Average ranked scores were used to generate the top 4 barriers and facilitators for inclusion in the survey, so as not to overburden participants. This group further advised on the wording of the final set of items (see Textbox 1).

**Final attributes and their levels used in the discrete choice experiment.**

1. Accuracy of detection
  High: detects symptoms correctly 75% of the time.
  Moderate: detects symptoms correctly 50% of the time.
  Low: detects symptoms correctly 25% of the time.
2. Privacy
  High: all information is stored on the device; no information leaves the device unless authorized by the user.
  Moderate: information that is hard to use to identify the user is automatically shared with the organization that makes the device or software.
  Low: information that can identify the user (eg, through digital identifiers or location) is automatically shared with the organization that makes the device or software.
3. Benefit to user
  High: clear, proven, practical benefit (ie, the device currently contributes to health management).
  Low: possible but unknown benefit (ie, the system is being tested as part of research and may contribute to health management now or in the future).
4. Scope for support
  High: personal use only (eg, self-management of a health condition).
  Low: personal use and ability to share identifiable information with a clinician (eg, clinician-assisted management of a health condition).

#### Survey Format and Scenario Development

**Figure 1:**
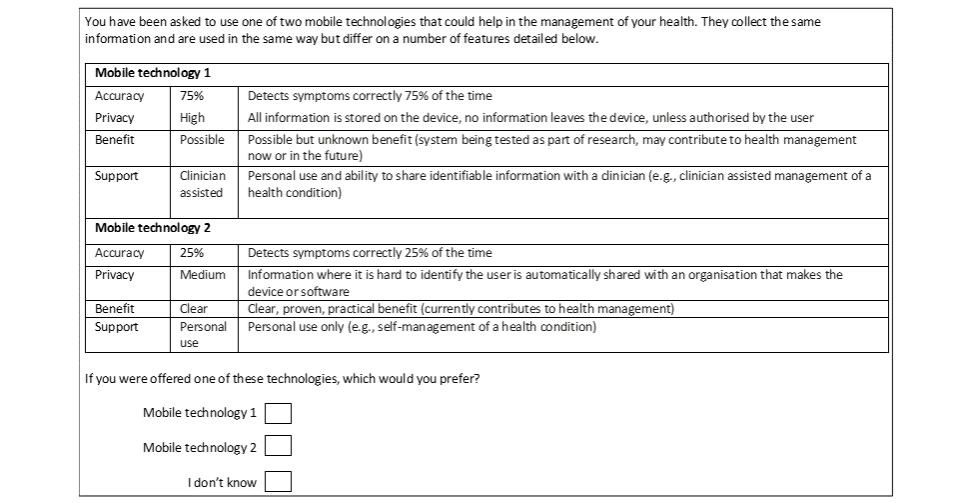
Questionnaire example.

The final 4 attributes and levels selected provide 2^2^×3^2^=36 unique combinations. We used NLOGIT software (NLOGIT) to generate an 8-task fractional-factorial main effects design, aiming to obtain near orthogonality, conducted in 1 block. We asked participants to choose between 2 different unlabeled mobile technology descriptions and an opt-out option (“I don’t know”). The scenarios were balanced in terms of the number of times that each level of the attribute appeared; Figure 1 shows an example. The survey was created using the software Qualtrics (Qualtrics Experience Management).

#### Additional Data Collected

The use and acceptance of mobile technology (eg, smartphones and wearable devices) were assessed, the latter with a modified version of the Unified Theory of Acceptance and Use of Technology 2 (UTAUT2) questionnaire, which provides a score from 0 to 28 [32] (details are described in Multimedia Appendix 1). Information on age, gender, level of education, self-reported diagnosis, and whether people had experienced an episode of a depressive disorder within the past 2 years were also collected.

#### Survey Administration and Data Collection

Participants were recruited from two sources to increase participant variation and overcome the digital divide: (1) through charities such as the MS Society and Epilepsy Action, who circulated an online link to the survey and promoted it on social media, and (2) through outpatient hospital clinics for people with epilepsy and MS, with the researchers facilitating survey access. No incentives were offered.

### Sample Size and Data Analysis

Formal sample size calculations are challenging for DCEs, but their level of precision increases rapidly up to 150 participants, [33] so we adopted this as our sample size guide for each group (ie, MS and epilepsy). Analysis was undertaken using a mixed multinomial logit model which allows for unobserved heterogeneity of preferences across respondents. This modeling approach also accommodates multiple observations per respondent and relaxes the assumption of independence of irrelevant alternatives required in the commonly applied multinomial logit model. Responses of “I don’t know” were treated as missing data rather than rejection of the device. It was hypothesized that higher levels of privacy, greater levels of support, increased accuracy of detection, and clearer benefit to the user would influence respondents’ decisions. Coefficient signs and significance were explored to investigate whether the data supported these hypotheses. The attribute accuracy of detection was specified as a categorical variable with 3 levels to allow for a nonlinear relationship with utility. All categorical variables were effects coded to aid interpretation [34]. Marginal rates of substitution were calculated to express the degree to which respondents would trade off attribute levels and accuracy. It was assumed that the change in accuracy was linearly related to the change in utility for changes in accuracy within the levels modeled.

The effects of 7 prespecified patient characteristics on response data were investigated. The characteristics were health condition (ie, MS or epilepsy), age, gender, education (categorized as low, medium, or high), depression within the past 2 years (categorized as yes or no), current user of wearable technology (categorized as yes or no), and score for acceptance of technology. Each patient characteristic was investigated separately by inclusion of interaction effects. Improvement in the Akaike information criterion was used as a criterion for a significant difference in preferences by subgroup. Interaction terms for all subgroups identified as having a significant impact on preferences were included in a single model. Backwards elimination was undertaken to assess whether the identified characteristics were capturing different underlying distributions of preferences across respondents. Characteristics were retained when inclusion of their interaction terms minimized the Akaike information criterion. The impact of these characteristics on preferences was quantified by recalculating the marginal rates of substitution by patient subgroup, utilizing a mixed logit model including interaction terms for that patient characteristic.

## Results

### Respondent Characteristics

A total of 318 respondents completed the survey. Of these, 141 (44%) of the respondents had MS, 175 (55%) had epilepsy, and 2 (0.6%) had both MS and epilepsy. All respondents answered at least one question. Table 1 reports the demographic and other characteristics of these respondents.

The coefficients from the mixed logit model excluding interactions with patient characteristics are shown in Table 2. Improved accuracy, higher privacy, increased level of benefit to the user, and the availability of clinical support were all associated with an increased likelihood of selecting a mobile technology device. Accuracy was the most important attribute, with a nonlinear effect; a move from low to moderate accuracy was valued higher than a move from moderate to high accuracy. The next most important attribute was privacy. Again, there was a modest nonlinear effect, with a stronger preference for moving from low to moderate privacy. Clinical support was the least valued of the attributes. The SDs reflect the impact of unobserved heterogeneity of preferences. There was evidence of unobserved heterogeneity in preferences for low versus moderate accuracy and for clinical support.

Table 3 reports the marginal rates of substitution for each attribute compared to accuracy. This is the percentage of accuracy that respondents were prepared to trade to achieve an improvement in the remaining attributes. Respondents were prepared to trade a modest amount of accuracy to achieve an improvement in privacy. Clinical support, in contrast, was not valued; respondents were only prepared to accept small reductions in accuracy in exchange for a high level of clinical support.

**Table 1:** Characteristics of the respondents (N=318) divided by the 7 variables included in the model.

Characteristics			Recruited through charities and social media		Recruited through hospital clinics		Total
**Health condition**							
	Epilepsy, n (%)	159 (89.8)		18 (10.2)		177 (55.7)	
	MS, n (%)	24 (16.8)		119 (83.2)		143 (45.0)	
Age, median (range)			46 (17-77)		40 (18-76)		44 (17-77)
Female, n (%)			128 (69.9)		96 (65.2)		217 (67.9)
**Education**							
	“A” level^a^ or equivalent, n (%)	50 (27.3)		37 (27.4)		87 (27.4)	
	Degree level, n (%)	81 (44.4)		66 (48.9)		147 (46.3)	
Positive for symptoms of depression within the past 2 years, n (%)			63 (34.4)		44 (32.6)		107 (33.6)
Current user of wearable technology, n (%)			62 (33.9)		36 (26.7)		98 (30.8)
Acceptance of technology, median (range)			0.8 (0.15-1)		0.7 (0.16-1)		0.7 (0.15-1)

^a^A-Levels are qualifications usually undertaken in the 12th and 13th year of school (up to age 18).

**Table 2:** The mixed logit model (no interactions with respondent characteristics).

Attribute	Coefficient (SE)	*P* value	95% CI
High accuracy	1.04 (0.06)	<.001	0.92 to 1.17
Low accuracy	–1.27 (0.07)	<.001	–1.41 to –1.13
High privacy	0.53 (0.05)	<.001	0.42 to 0.63
Low privacy	–0.66 (0.06)	<.001	–0.78 to –0.54
Benefit	0.37 (0.04)	<.001	0.30 to 0.44
Clinical support	0.18 (0.03)	<.001	0.11 to 0.24

**Table 3:** Percentage of accuracy respondents were willing to trade (mixed multinomial logit model, N=318).

Attribute	Acceptable change in accuracy from high to moderate	Acceptable change in accuracy from moderate to low
For high privacy	13%	10%
For moderate privacy	16%	13%
For high benefit	9%	7%
For high clinical support	4%	4%

### Subgroup Analyses

The coefficients from the mixed logit model including interactions with patient characteristics can be found in Table 4 and Table 5. Regression analyses indicated that health condition (ie, MS or epilepsy), age, gender, and depression had no significant effect on preferences. Current use of wearable technology had a marginal impact on preferences. Technology acceptance did have an impact, as did education level. After a multivariate analysis that included wearable technology use, education level, and technology acceptance score, wearable technology use was no longer significant. Preferences varied significantly for patients in the low education group but not between those in the average or high education groups. Table 6 reports the marginal rates of substitution for each attribute compared with accuracy for respondents with education beyond age 18 compared to those without, and for patients with a high technology acceptance score (in the 75th percentile) compared to those with a low score (in the 25th percentile).

Respondents with lower technology acceptance scores were prepared to trade far more accuracy for an improvement in privacy. The impact of technology acceptance on preferences for clinical benefit and clinical support was more modest. Respondents with high technology acceptance were prepared to trade slightly more in terms of loss of accuracy to improve clinical benefit or clinical support. These data suggest privacy is a far greater concern for respondents with a low technology acceptance score. The impact of education was more modest. Respondents with low education appeared less inclined to trade accuracy for improvements in clinical benefit and privacy but were more prepared to trade accuracy for improvements in clinical support.

**Table 4:** Mixed logit model including interactions with technology acceptance.

Attribute	Coefficient (SE)	*P* value	95% CI
High accuracy	1.10 (0.26)	<.001	0.64 to 1.56
Low accuracy	–1.71 (0.26)	<.001	–2.22 to –1.20
High privacy	1.52 (0.22)	<.001	1.08 to 1.95
Low privacy	–1.45 (0.24)	<.001	–1.91 to –0.99
Benefit	0.37 (0.14)	.007	0.10 to 0.63
Clinical support	0.19 (0.13)	.13	–0.05 to 0.44
Tech acceptance*high accuracy	0.01 (0.32)	.98	–0.61 to 0.62
Tech acceptance*low accuracy	0.52 (0.34)	.13	–0.15 to 1.19
Tech acceptance*high privacy	–1.32 (0.28)	<.001	–1.88 to –0.76
Tech acceptance*low privacy	1.07 (0.31)	.001	0.46 to 1.68
Tech acceptance*benefit	0.03 (0.18)	.88	–0.33 to 0.38
Tech acceptance*clinical support	0.00 (0.17)	.99	–0.34 to 0.34

**Table 5:** Mixed logit model including interactions with technology acceptancea.

Attribute	Coefficient (SE)	*P* value	95% CI
High accuracy	1.10 (0.07)	<.001	0.96 to 1.25
Low accuracy	–1.37 (0.08)	<.001	–1.53 to –1.20
High privacy	0.58 (0.07)	<.001	0.45 to 0.71
Low privacy	–0.70 (0.07)	<.001	–0.85 to –0.56
Benefit	0.41 (0.04)	<.001	0.33 to 0.50
Clinical support	0.18 (0.04)	<.001	0.11 to 0.26
Low education*high accuracy	–0.21 (0.13)	.01	–0.47 to 0.04
Low education*low accuracy	0.30 (0.14)	.04	0.02 to 0.59
High education*high privacy	–0.15 (0.11)	.19	–0.37 to 0.07
Low education*low privacy	0.13 (0.13)	.32	–0.13 to 0.39
Low education*benefit	–0.16 (0.08)	.05	–0.31 to 0.00
Low education*clinical support	–0.02 (0.08)	.82	–0.17 to 0.13

^a^The IQR was used to define high and low technology acceptance scores.

**Table 6:** Percentage of accuracy respondents were willing to trade, divided by education level and technology acceptance.

Attribute	Education beyond age 18		No education beyond age 18			Low technology acceptance score (0.554)^a^			High technology acceptance score (0.875)^b^	
	Acceptable change in accuracy from high to moderate	Acceptable change in accuracy from moderate to low	Acceptable change in accuracy from high to moderate	Acceptable change in accuracy from moderate to low	Acceptable change in accuracy from high to moderate		Acceptable change in accuracy from moderate to low	Acceptable change in accuracy from high to moderate		Acceptable change in accuracy from moderate to low
For high privacy	13%	11%	12%	10%	18%		14%	8%		7%
For moderate privacy	16%	13%	16%	13%	19%		15%	11%		10%
For high benefit	9%	8%	7%	6%	9%		7%	9%		8%
For high clinical support	4%	3%	5%	4%	4%		3%	4%		4%

^a^The value for low technology acceptance represents the 25th percentile.

^b^This value for high technology acceptance represents the 75th percentile.

### Sensitivity Analysis

Including missing data as an active decision to reject both technologies for the respective question (ie, an “opt-out”) had a minimal impact on the magnitude of the model coefficients, and their significance and direction were unchanged. These results are available on request.

## Discussion

### Principal Findings

**Figure 2:**
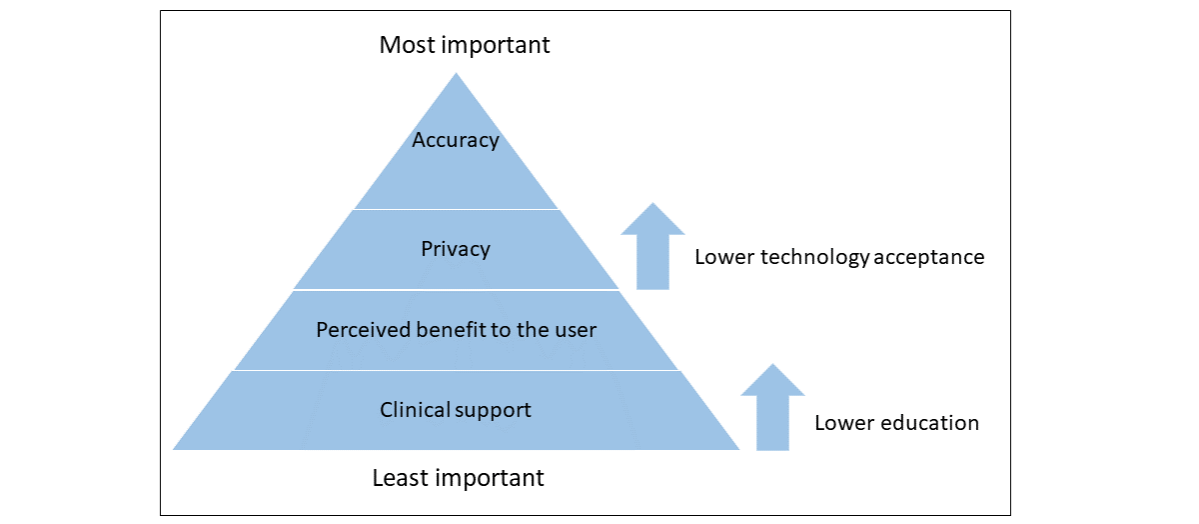
A hierarchy of factors to consider in the design of mobile technologies to influence engagement for people with a neurological condition, with the size of each segment indicating the weight of the preference. The arrows indicate potential moderating factors: preferences for privacy and clinical support increased for individuals with lower technology acceptance and lower education, respectively.

Our analyses revealed that people with epilepsy or MS value higher levels of privacy, greater levels of support, increased accuracy, and a clearer benefit to the user when selecting mHealth technology devices. This is a key finding that is in line with our hypotheses and validates feedback received from people with epilepsy and MS in other studies [1,5,6]. Of all these factors, people preferred a higher degree of accuracy, regardless of their diagnosis. When asked to make compromises between levels of privacy, clinical support, accuracy, and benefit to the user, people were willing to trade some accuracy for greater privacy but were less influenced by the other factors. This analysis reveals a hierarchy in the importance of factors influencing engagement with mobile technologies (Figure 2).

We wanted to explore individual variability in how people traded off these attributes, because this should lead to a more tailored or personalized approach to RMT development. We found no evidence that age, gender, or experience of depression affected preferences. This is in contrast to other studies, such as one that found that age influences technology use in a different patient population [11]. We did, however, discover the following: (1) the people who were less accepting of technology placed greater value on privacy and were willing to give up some degree of accuracy for privacy, and (2) people with no qualifications beyond those that might have been obtained before the age of 18 were willing to compromise some degree of accuracy to receive greater clinical support. These moderating factors are shown in Figure 2.

This model will help those designing mobile technologies to prioritize features for development that can maximize engagement. Accuracy is the key feature; individuals were willing to make compromises on accuracy, but the reduction in accuracy they were prepared to accept for improvements in other characteristics was relatively small (<20%). We did not find that people with MS and people with epilepsy had different preferences; however, accuracy may have a different meaning for devices that detect seizures in epilepsy and devices that measure symptom recurrence or deterioration in MS. Issues to do with privacy and the willingness to share information with a clinician were more transdiagnostic, with subgroup differences relating more to trust and familiarity with technology and educational level. This indicates that some level of personalization may be required in the design of devices.

### Strengths and Limitations

This study is the first of its kind to try to understand the relative value and influences of factors affecting engagement with mobile technologies for people with neurological conditions. It used a quantitative approach, which is novel in this area of research, adopted from the field of health economics. The study size was large enough to explore some subgroups, selected for their hypothesized relationship with technology use, but future researchers may wish to focus on a greater number of health-related variables, such as illness severity, which we were unable to discuss in this paper. Basing our study on the completion of an online survey, albeit one that was supervised for participants who were recruited from clinics, may have led to a sampling bias toward respondents who were more familiar with technology. However, there were few differences in the patterns of the data and in the makeup of the samples. An additional limitation is that we did not design the DCE in a way to allow validity checks on choice data, such as by including repeated tasks or dominant alternatives.

### Conclusions

We have shown that people with epilepsy and MS are influenced by factors such as the accuracy, privacy, benefit of the technology, and the amount of clinical support received, but that in some instances they are willing to make compromises. These preferences should be factored into the design of mHealth technologies, alongside the views of other stakeholders, in the future.

## Appendix

Multimedia Appendix 1 Modified UTAUT2 survey.

## Abbreviations

DCE: discrete choice experiment
MS: multiple sclerosis
RMT: remote measurement technology
