# Supplementary material for: Key Drivers and Facilitators of the Choice to Use mHealth Technology in People With Neurological Conditions: Observational Study
Source: JMIR Form Res. 2022 May 23;6(5):e29509. doi: 10.2196/29509 (PMC9171601; doi:10.2196/29509)
Supplement: Multimedia Appendix 1 [file formative_v6i5e29509_app1.docx]

Modified UTAUT2 survey

Performance expectancy (PE)

PE1: I would find a wearable device useful in my daily life.

PE2: using a wearable device would help accomplish things more quickly.

PE3: using a wearable device would improve the quality of my daily healthcare seeking.

Hedonic motivation (HM)

HM1: using a wearable device would be fun.

HM2: using a wearable device would be enjoyable.

HM3: using a wearable device would be entertaining.

Effort expectancy (EE)

EE1: learning how to use a wearable device would be easy for me.

EE2: I will find a wearable device easy to use.

EE3: It would be easy for me to become skilful at using a wearable device.

Social influence (SI)

SI1: people who are important to me would think that I should use a wearable device.

SI2: people who influence me would think that I should use a wearable device.

SI3: people whose opinions are valued to me would prefer that I use a

wearable device.

Functional congruence (FC)

FC1: the wearable device should be comfortable.

FC2: the wearable device should be fashionable.

FC3: the wearable device should be priced appropriately considering their quality.

Self-efficacy (SE)

SE1: it would be easy for me to self-monitor my health condition by using a wearable device.

SE2: I have the capability to use a wearable device to self-monitor my health condition.

SE3: I would be able to use a wearable device to self-monitor my health condition without much effort.

Perceived risk (PPR)

PR1: it would be risky to disclose my personal health information to those providing the wearable device.

PR2: there would be high potential for loss associated with disclosing my personal health

information to those providing the wearable device.

PR3: there would be too much uncertainty associated with giving my personal health

information to those providing the wearable device.

Behavioral intention (BI)

BI1: I intend to use a wearable device in the future.

BI2: I intend to use a wearable device at every opportunity in the future.

BI3: I plan to increase my use of a wearable device in the future.

Trust in medical researchers (TMR)

TMR1: Doctors who do medical research care only about what is best for each patient

TMR2: Doctors tell their patients everything they need to know about being in a research study

TMR3: Medical researchers treat people like “guinea pigs”

TMR4: I completely trust doctors who do medical research
